# Improving gastric cancer care in Brazil: the ABCG (Brazilian Gastric Cancer Association) commitment and call to action

**DOI:** 10.1590/0102-672020260000022e1951

**Published:** 2026-08-21

**Authors:** Andre Maciel da SILVA, Marcus Fernando Kodama Pertille RAMOS, Antonio Carlos WESTON, Paulo KASSAB, Ulysses RIBEIRO-JUNIOR, Nora Manoukian FORONES

**Affiliations:** 1 Associação Brasileira de Câncer Gástrico – São Paulo (SP), Brazil.

 Gastric cancer remains an important cause of morbidity and mortality in our country^4^. Surgical resection has been the cornerstone of curative treatment, while the incorporation of new therapeutic modalities, particularly systemic chemotherapy, has contributed to improved patient survival^1^. Nevertheless, major management challenges persist, including late diagnosis, with nearly half of patients presenting with stage IV disease at diagnosis, limited access to contemporary chemotherapy regimens, and insufficient availability of treatment in specialized centers capable of providing integral, comprehensive multidisciplinary care^2,3^. 

 The Brazilian Gastric Cancer Association (ABCG), founded in 1999, brings together specialists involved in the treatment of gastric cancer in Brazil, with a strong focus on continuing medical education and professional training^4^. As part of its activities, the Association organizes an annual scientific meeting. During the 2025 meeting, a “Commitment Letter” was drafted and formally delivered to the Secretary of Health of the city of Rio de Janeiro, Mr. Daniel Ricardo Soranz Pinto, outlining key demands and strategic actions considered necessary to improve gastric cancer outcomes in Brazil. 

 This initiative was the first ABCG’s advocacy effort in this context. In this editorial, we share the contents of this Commitment Letter to broaden awareness within the Brazilian surgical and oncological community. We believe that, in addition to their educational and scientific roles, medical associations should advocate for patients and actively engage with health authorities to support policies that improve cancer care. We hope this initiative is seen as an example to be followed by other national medical societies. 

## COMMITMENT LETTER –– RIO DE JANEIRO, SEPTEMBER 29, 2025

 “Considering the high incidence and lethality of gastric cancer in Rio de Janeiro and in Brazil, being the fourth most frequent malignant neoplasm among men and the sixth among women; 

 Considering the difficulties in implementing a policy for gastric cancer prevention, diagnosis, and therapeutic care in Rio de Janeiro and in Brazil; 

 Considering the difficulties in articulating the oncology care network in Rio de Janeiro, particularly regarding the mechanisms of patient referral and counter-referral; 

 We, members of civil society and committed to working together with the public authorities, have prepared this Commitment Letter to support public health policies for gastric cancer care:

1. The prevention of gastric cancer begins with healthy lifestyle habits, including adequate nutrition (rich in vegetables, greens, fruits, fibers, and fish, moderate in red meat and salt, and low in canned foods, fried foods, and processed products), physical exercise, smoking cessation, minimal alcohol consumption, combating obesity since childhood, and eradication of the *Helicobacter pylori* bacterium (the main cause of gastric cancer in our setting). These measures should be encouraged to the population and disseminated through primary healthcare units and community health agents. They should also be incorporated into the daily routine of public schools (school meals, public cafeterias, physical education classes, and lectures about the harms of smoking) at the three levels of government;
2. Aware of the close correlation between *Helicobacter pylori* and dyspeptic diseases/gastric cancer, expanding basic sanitation and providing better quality water to the entire population of Rio de Janeiro State and Brazil are fundamental. Once the bacterium in the stomach is detected, its eradication becomes mandatory, especially in the pediatric and adolescent population, in which the greatest correlation between eradication and gastric cancer prevention is observed. We also propose the development of a vaccine against *Helicobacter pylori* by FIOCRUZ, a renowned federal research institute located in Rio de Janeiro;
3. Access to upper gastrointestinal endoscopy is key to preventing *Helicobacter pylori* detection) and for the earliest possible diagnosis of gastric cancer after the onset of the first symptoms. Considering that the symptoms of dyspeptic syndromes resemble the initial symptoms of gastric cancer, we propose a policy of EVERY DYSPEPSIA = ENDOSCOPY, since this would provide the population with a more accurate diagnosis of dyspeptic diseases, detection of *Helicobacter pylori*, and earlier diagnosis of gastric cancer;
4. We observe a significant delay in the release of histopathological reports (HPR) from biopsies of suspected gastric cancer lesions. This delays diagnosis confirmation and, consequently, treatment of the disease. Therefore, we propose a protocol for releasing the HPR within 15 days after biopsy and the inclusion of a mandatory immunohistochemical panel including HER-2, DNA repair genes (microsatellite instability), PD-L1, and Claudin 18.2, in view of recent therapeutic advances in gastric cancer (immunotherapy and targeted therapy);
5. Once gastric cancer is confirmed, referral from the primary healthcare unit to the high-complexity center must occur rapidly, in accordance with Federal Law 12.732/2012, known as the “Sixty-Day Law”. This law establishes that the patient must START ONCOLOGICAL TREATMENT within sixty days after the diagnosis of gastric cancer (date the HPR was released);
6. Primary and secondary healthcare units in ALL municipalities of Rio de Janeiro/Brazil, working in collaboration with the local and/or regional technological infrastructure, must be prepared to provide diagnosis and initial staging of gastric cancer, including upper gastrointestinal endoscopy with biopsy, contrast-enhanced computed tomography (chest, upper abdomen, and pelvis), and laboratory tests, including tumor markers;
7. Gastric cancer treatment must be CENTRALIZED, so that High-Complexity Oncology Units (UNACONs) and High-Complexity Oncology Centers (CACONs) function as referral hospitals for a given regional group of municipalities, which would facilitate patient flow in the referral and counter-referral process;
8. Referral units must be structured to provide COMPREHENSIVE treatment for patients with gastric cancer, including surgery, chemotherapy, immunotherapy, targeted therapy, radiotherapy, endoscopic treatment, and palliative care. Intraoperative frozen section analysis is a fundamental procedure in gastric cancer surgery and should be included in the list of mandatory procedures;
9. Referral units in Rio de Janeiro and other Brazilian states (UNACONs and CACONs) must be INTEGRATED, developing therapeutic protocols, research lines, and educational activities (forums, symposia, and congresses), thereby contributing to technological and academic advancement at the state and national levels. We are severely lacking in research and technology development in oncology, including gastric cancer;
10. It is essential to establish a public career plan in the field of oncology care that is attractive to the professionals involved. Currently, we are witnessing a wave of “brains lost” to the private sector due to hostile salary conditions and inadequate hospital infrastructure within the public healthcare network. It is important to remember that 75% of the Brazilian population is treated by the Brazilian Unified Healthcare System (SUS)."

## Data Availability

The datasets generated and/or analyzed during the current study are available from the corresponding author upon reasonable request.
